# Supplementary figures and images for: Transcriptomic Studies of Antidepressant Action in Rodent Models of Depression: A First Meta-Analysis
Source: Int J Mol Sci. 2022 Nov 4;23(21):13543. doi: 10.3390/ijms232113543 (PMC9654684; doi:10.3390/ijms232113543)

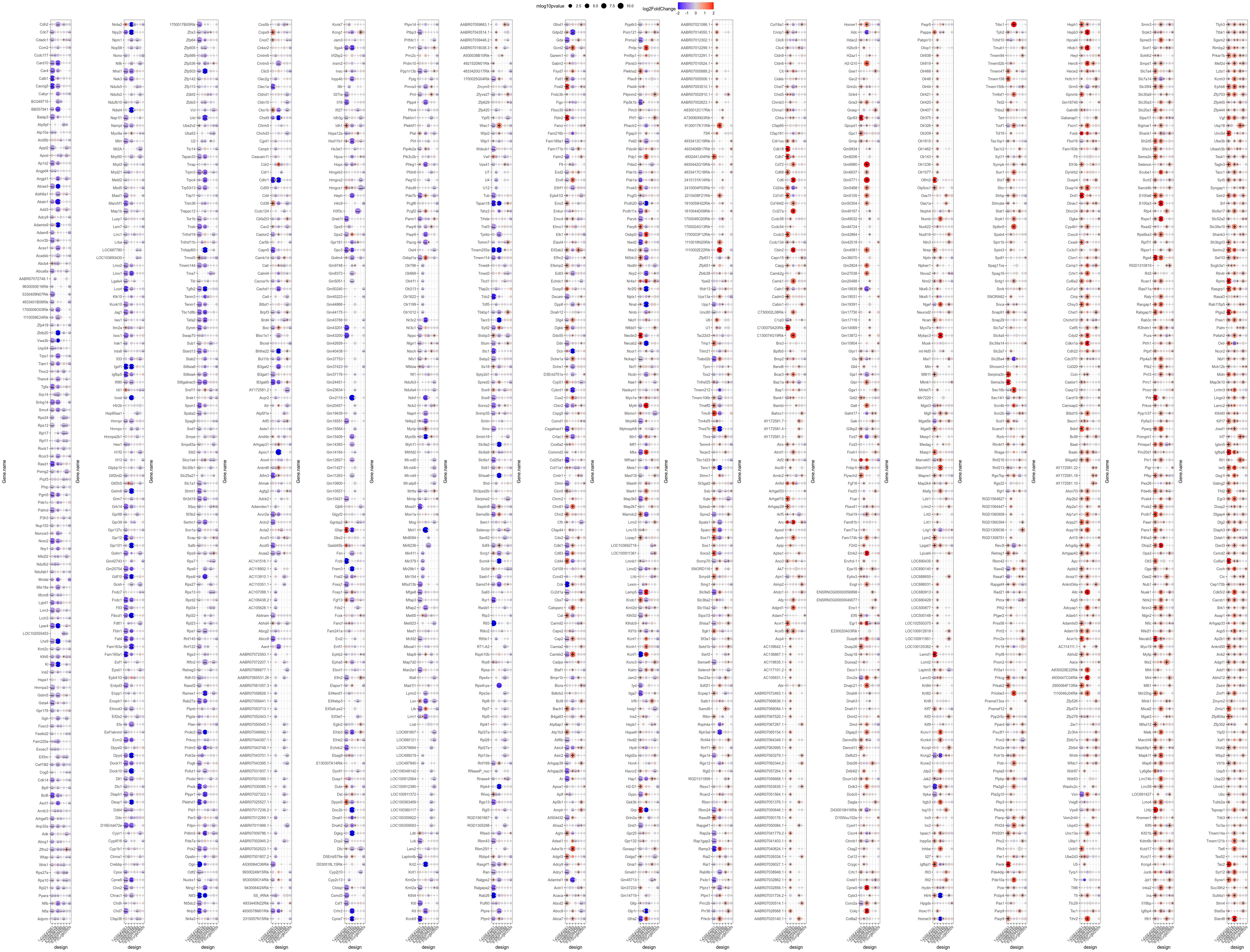

Supplement: Supplementary file 1 [file ijms-23-13543-s001.zip › Supplementary Figure S1.png]

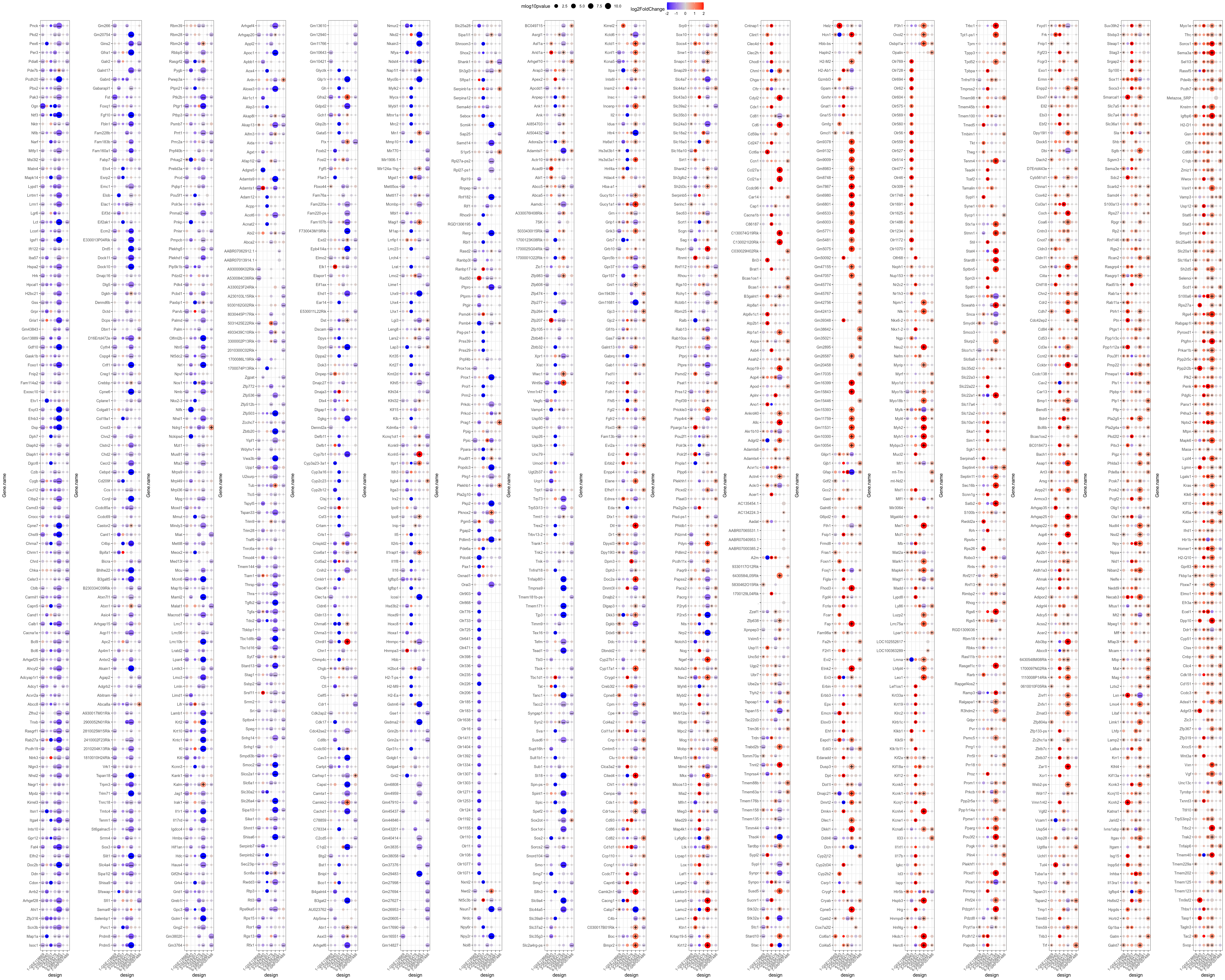

Supplement: Supplementary file 1 [file ijms-23-13543-s001.zip › Supplementary Figure S2.png]
